# Companion Animal Relationships and Adolescent Loneliness during COVID-19

**DOI:** 10.3390/ani11030885

**Published:** 2021-03-19

**Authors:** Megan K. Mueller, Amanda M. Richer, Kristina S. Callina, Linda Charmaraman

**Affiliations:** 1 Department of Clinical Sciences, Cummings School of Veterinary Medicine, Tufts University, North Grafton, MA 01536, USA; 2 Jonathan M. Tisch College of Civic Life, Tufts University, Medford, MA 02155, USA; 3 Wellesley Centers for Women, Wellesley College, Wellesley, MA 02481, USA; aricher@wellesley.edu (A.M.R.); lcharmar@wellesley.edu (L.C.); 4 Lynch Research Associates, Natick, MA 01760, USA; kristina.callina@gmail.com

**Keywords:** human-animal interaction, pet ownership, loneliness, adolescence, COVID-19

## Abstract

**Simple Summary:**

This study assessed the relationship between pet ownership, pet attachment, loneliness, and coping with stress before and during the COVID-19 pandemic. Contrary to our hypotheses, results did not support the presence of a buffering effect of pet ownership on loneliness, with pet ownership predicting increases in loneliness from pre-pandemic to during the pandemic. Dog owners showed lower levels of loneliness prior to the pandemic as well as higher levels of attachment, suggesting possible species-level differences in these relationships. Pet owners also reported spending time with their pet as a highly used strategy for coping with stress, suggesting that future research should explore the role of pets in coping with stress and social isolation during the pandemic. These results indicate that the relationship between pet ownership and adolescent loneliness during the pandemic is complex and warrants further research.

**Abstract:**

The pandemic associated with the emergence of the novel coronavirus (COVID-19) is an unprecedented historical event with the potential to significantly impact adolescent loneliness. This study aimed to explore the role of companion animals and attachment to pets in the context of the pandemic. We used longitudinal quantitative survey data collected prior to and during the pandemic to assess the role of pets in predicting adolescent loneliness. Pet ownership was not a significant predictor of loneliness before the pandemic, but did predict higher levels of loneliness during COVID-19 as well as higher increases in loneliness from before to during the pandemic. Dog ownership predicted lower levels of loneliness prior to, but not during the pandemic, and dog owners were significantly more attached to their pets than non-dog pet owners. Adolescents with pets reported spending more time with their pets during the pandemic, and frequently reported pet interactions as a strategy for coping with stress. Overall, the results from this study did not support the presence of a buffering effect of companion animals on loneliness for adolescents and indicate complexity in the relationships between pet ownership, attachment, loneliness, and coping with stress. These results suggest a need for additional research further assessing how features of the relationship such as species and relationship quality might contribute to adolescent mental health outcomes.

## 1. Introduction

The global pandemic associated with the emergence of the novel coronavirus (COVID-19) is an unprecedented historical event with potential effects on human mental health. Initial research on the COVID-19 pandemic has suggested that there are perceived impacts on quality of life, health, and emotional well-being for both adults and children [1,2,3]. While the psychological consequences of COVID-19 are relevant across the lifespan, there is significant risk to child and adolescent mental health in particular [2]. With a lack of in-person human-to-human social interaction due to social distancing, it is important to explore the role of relationships with companion animals in providing social support and buffering loneliness [4]. As more research emerges assessing human-pet relationships during the pandemic, there is a need for examining these relationships specifically in the context of adolescent loneliness and stress.

Youth are uniquely impacted by COVID-19-related lockdowns due to the disruptions caused by school closures, which includes lack of access to mental health support and peer interactions [5]. Adolescence is a developmental period marked by significant social development, and peer relationships are a critical component of how youth access social support [6]. Even outside of the context of a pandemic, individuals under the age of 25 demonstrate higher levels of loneliness than many other age groups, and greater time spent in social engagement is a protective factor against loneliness [7]. Therefore, loneliness and associated stressors are a significant concern for adolescents during lockdowns and community closures due to limits on social engagement. Lack of access to peers may put youth at risk for poor mental health outcomes in an already stressful time [8].

One potentially important source of emotional support during this challenging period may be a relationship with a pet. Research has demonstrated that strong social relationships outside of the school setting can provide a buffer for some of the social-emotional effects of socially challenging time periods [9]. Pets provide stable social contact and positive social interactions. Youth frequently report turning to their pets for emotional support and comfort when distressed [10], and a majority of pet owners consider their pets to be family members [11]. Acquiring a dog has been associated with reduced loneliness [12,13], and there is some initial evidence that adolescents who own pets are less lonely than non-pet owners [14]. When access to face-to-face human social support is restricted due to the pandemic, pet owners may rely on their pets to supplement these interactions [4] and to diminish social isolation [15]. However, these findings are complex; for example, Powell et al. [13] found that adjusting for covariates such as education level nullified the effects of dog acquisition on loneliness. In addition, the majority of existing research to date on pets during the pandemic has focused on adults [1,15,16,17], with a range of mixed findings across samples and outcomes. To complement these initial studies, there is still a need for complementary research assessing similar relationships in youth.

Specifically, quality of pet relationship is likely an important factor in predicting the protective contribution of companion animals. Attachment to a pet can serve as an emotional buffer during times of stress and has been associated with the utilization of adaptive social coping skills [18]. A relationship with an animal can become part of an adolescent’s repertoire of social resources, which may reduce the perception of social risk and lead to decreased anxiety. Underscoring this point, perceived social support from a pet has been associated with higher levels of pet attachment [19]. There is some evidence that pet attachment is strongest in early adolescence, and decreases with age [20], highlighting the importance of pet attachment for this age group. Pet relationships can also provide a way for youth to process their emotions during times of stress as a “safe” outlet for emotional disclosure. When lack of peer contact leads to loneliness, an adaptive relationship with an animal may provide the emotional support that a youth needs in order to cope with this change in peer social support without increasing anxiety. During the pandemic, pets may reduce uncertainty by helping youth keep a consistent routine and providing a source of predictability [4].

Initial research on companion animal interactions during COVID-19 has suggested that some pet owners perceive that their pet has helped them during periods of confinement [1]. In some cases, pet ownership appeared to provide a protective effect, where individuals with pets had smaller decreases in mental health and smaller increases in loneliness as a result of the pandemic [17]. However, pet interactions were also associated with poorer mental health indices prior to the pandemic [17], and in some cases did not predict loneliness [16].

The emerging research suggests that there may be some species differences in these effects; several studies found protective relationships between dog ownership and loneliness [16] and quality of life [1]. However, these findings often did not replicate across all species (with some specific differences between dog and cat owners [16]). These species differences align with existing research on adolescent-pet relationships, which suggested that dog owners report higher levels of satisfaction, companionship, and attachment [21,22], as well as other research with adult populations [23]. Therefore, exploring the unique effects of youth-dog relationships is an important facet of understanding if and how pets may ameliorate loneliness during COVID-19.

Given the potentially significant social challenges that adolescents may face during COVID-19, it is important to explore the role of pets and pet attachment in providing social support outside of the school setting. This study aimed to contribute to the emerging research on companion animals and mental health during the pandemic by assessing the relationships between pet ownership, pet attachment, loneliness, and stress coping for adolescents both before and during the COVID-19 pandemic. Using data from a larger longitudinal study of adolescent development in youth ages 10–16 that was collected both pre-COVID-19 and during COVID-19, we addressed: (1) If the frequency and nature of adolescent-pet interactions changed from pre-COVID-19 to during the pandemic, (2) if there was a relationship between pet ownership and/or pet attachment and adolescent loneliness before and during COVID-19, (3) how adolescents incorporated their pets into strategies for coping with stress, and (4) if there were any differences between dog owners and non-dog pet owners regarding the relationship between loneliness and interactions with pets.

## 2. Materials and Methods

### 2.1. Procedure

This study used data collected as part of a larger longitudinal study of early adolescent social technology use [24,25]. Middle schools in the Northeastern US were recruited in 2019 based on varying school enrollment size, internet accessibility, and diverse racial/ethnic composition (e.g., a minimum of 30% of students identifying as a racial/ethnic minority). Upon obtaining Institutional Review Board (IRB) approval from our institution and school district-level permissions, we worked with school and afterschool liaisons to distribute informed consent/opt-out forms (in English, Spanish, and Portuguese) to parents through paper flyering, parent email listservs, school electronic newsletters, and direct emails. For the pre-COVID-19 pandemic data collection (October–December 2019, referred to as Time 1), members of the research team proctored the online Qualtrics survey in person during a pre-scheduled advisory period lasting up to 60 min. Students primarily used school or afterschool program-provided Chromebooks and the survey took about 25–40 min to complete. Since survey links were emailed to students through their Google Classroom, those who were absent during the survey administration were still able to participate from home.

During the COVID-19 pandemic, we attempted to collect data from the same cohort in June–July 2020 (referred to as Time 2). Due to schools conducting remote learning at the time, we relied on different strategies to retain our sample. These included: (a) principals and afterschool program directors sending out the parent informed consent/opt out forms through email and parent listservs, (b) the research team using contact information provided by the earlier cohort (when available) to re-engage them in completing the Time 2 survey, and (c) offering raffle prize gift cards to increase engagement. In only 2 out of the 3 sites, students had access to school-provided Chromebooks to complete their surveys from home.

Participation rates for Time 1 in October–December 2019 (pre-COVID-19 pandemic) ranged from 40% in the afterschool programs to 93% in the whole school data collection, which includes 5.2% parent or student opt-out or absence on the day the survey was administered. Participation rates for Time 2 in June–July 2020 (COVID-19 pandemic period) ranged from 15% in the afterschool programs to 47% in the whole school data collection, which includes 16.3% parent or student opt-out. Due to the remote learning period caused by the COVID-19 pandemic, it was difficult to capture an accurate rate of students absent during the survey data collection period as students were working asynchronously from home. For both data collection periods, an honorarium was provided to schools for participating as well as gift card incentives to teachers and program coordinators to help with survey recruitment. Students who participated in the survey were given an embossed pen from our institution, snacks (only during in-person afterschool programming), and were entered into a raffle prize for a chance to win a $25 gift card.

### 2.2. Participants

As previously noted, Time 1 data collection occurred prior to COVID-19 (*n* = 1033) and Time 2 occurred during COVID-19 (*n* = 357). At Time 1, the average age of participants was 12.69 years (*SD* = 1.21); 50% were female, 49% were male, and 1% identified as other gender. Forty-nine percent self-identified their race/ethnicity as White, 18% Hispanic, 10% Black, 7% Asian, 2% as Native American, 5% as Biracial, and 10% as Other. At Time 1, 23% of participants reported receiving free or reduced-price lunch (proxy for socioeconomic status). At Time 2, the average age of participants was 13.02 years (*SD* = 1.06), 65% were female, 34% were male, and 1% identified as other gender. Sixty-three percent self-identified their race/ethnicity as White, 14% Hispanic, 8% Black, 8% Asian, 6% as Biracial, and 1% as Other. At Time 2, 20% of participants reported receiving free or reduced-price lunch.

In addition to the cross-sectional analyses, for analyses assessing change from Time 1 to Time 2, we use a subsample of 318 participants with data at both time points. Within this subsample, 65% were female, 34% were male, and 1% identified as other gender. Sixty-three percent self-identified their race/ethnicity as White, 14% Hispanic, 8% Black, 8% Asian, 7% as Biracial, and 1% as Other. Nineteen percent reported receiving free or reduced-price lunch. Within the matched sample, average age was 12.40 years (*SD* = 1.07) at Time 1, and 13.00 years (*SD* = 1.04) at Time 2.

### 2.3. Measures

#### 2.3.1. Pet Ownership

Pet ownership was measured using a single yes or no item asking youth whether they had a pet. If participants indicated having a pet, they were asked about the species. Responses included dog, cat, reptile, fish, horse, other animal or other. For youth with multiple pets, they were asked to report the species of their favorite pet.

Average daily time spent with a pet was measured using a single item asking youth how much they spend with their favorite pet on a typical day. Responses ranged from “Less than 10 min per day” (1) to “6 h or more” (7).

#### 2.3.2. Pet Attachment

Pet attachment was measured using nine items from the Network of Relationships Inventory-Pet (NRI-Pet), which is validated for use with adolescents [21]. Items from the satisfaction, companionship, and disclosure domains were used, which measured satisfaction with pet relationship, how often they go places and enjoy doing things with their pet, spend time with their pet, how much they play around and have fun with their pet, how often they confide to their pet about secrets, feelings and things others don’t know, and how much they talk to their pet about everything. Items were rated on a 6-point scale ranging from “Little to none” (0) to “The most” (5). The overall pet attachment scale demonstrated excellent reliability; Time 1 α = 0.842, Time 2 α = 0.839. The average pet attachment for all pet-owning participants in the longitudinal subsample (*n* = 179) at Time 1 was 3.18 (*SD* = 0.99) and at Time 2 (*n* = 174) was 3.36 (*SD* = 0.95). The average pet attachment for all pet-owning participants (*n* = 555) at Time 1 was 3.19 (*SD* = 0.97) and for all pet owners (*n* = 192) at Time 2 was 3.36 (*SD* = 0.95). Scales at Time 1 and Time 2 were normally distributed.

#### 2.3.3. Loneliness

Loneliness was measured using a three-item scale asking students how often they feel left out, lack companionship, or isolated from others [26]. Items were measured on a 3-point scale ranging from “Hardly ever” (1) to “Often” (3), and demonstrated excellent reliability; Time 1 α = 0.800, Time 2 α = 0.787. The average loneliness for all students in the longitudinal subsample (*n* = 303) at Time 1 was 1.49 (*SD* = 0.56) and at Time 2 (*n* = 280) was 1.56 (*SD* = 0.57). The average loneliness for all students (*n* = 930) at Time 1 was 1.45 (*SD* = 0.52) and for students (*n* = 314) at Time 2 was 1.56 (*SD* = 0.57). Loneliness scales at Time 1 and Time 2 were normally distributed.

#### 2.3.4. Coping with Stress

At both time points youth indicated what strategies they used to cope with stress, including: being alone; spending time with family, friends, and/or pets; posting on social media; watching favorite movies or shows; exercising or sports; playing video or online games; or spending time outdoors or in nature. An additional two items, creating video content for social media and video hangouts, were added at Time 2. These items were reported on a 4-point scale ranging from “Mostly Disagree” (1) to “Mostly Agree” (4). Students could also select “Does not apply to me” for any of the above strategies.

### 2.4. Data Analysis

For cross-sectional analyses assessing Time 1 (pre-COVID-19) and Time 2 (during COVID-19) separately, the full cross-sectional samples were used. For analyses involving change from pre-COVID-19 to during-COVID-19, we used the matched sample of 315 participants with data at both time points. For comparisons by species, the pet type variable was dichotomized into dog owners and non-dog owners.

Item nonresponse ranged from 1% to 15% at both time points. We assessed if there were differences between participants with and without missing data at both time points. At Time 1, participants with missing data were more likely to receive free/reduced price lunch, and younger (as measured by grade level). There were no systematic differences regarding missing data at Time 2, indicating that data are missing at random at Time 2. Missing data by item is reported in the Supplementary Materials.

Descriptive statistics and frequencies are reported for pet ownership. Paired samples *t*-tests were used to assess change from Time 1 to Time 2 regarding time spent with a pet. Missing data for *t*-tests were handled using pair-by-pair deletion. We used regression models to assess the role of pet ownership, dog ownership and pet attachment in predicting loneliness, as well as species of pet predicting pet attachment and time with pet as a stress-coping strategy, controlling for gender, age, and free/reduced price lunch eligibility (proxy for socioeconomic status). All data assumptions were met for linear regression, including normality, linearity, independence, homoscedasticity, and no multicollinearity. We did not identify any outliers. Missing data in regression was handled using listwise deletion. Gender was dichotomized to male (0) or female (1) and other gender was coded as missing. Age was treated continuously and ranged from 10–16 years at Time 1 and 11–18 years at Time 2. Free/reduced price lunch was dichotomized as “yes” or “no or don’t know.” Pearson correlations were used to assess the relationship between time spent with a pet and pet attachment. Independent samples *t*-tests were used to assess differences between pet owners and non-pet owners on stress coping strategies. All analyses were conducted using Statistical Package for the Social Sciences (SPSS) version 26. Power analyses were conducted in G*Power 3.1.9.7., indicating that within the matched sample of 318 youth, we have the power of 0.80 to detect effects as small as 0.14 between time points. The threshold for statistical significance was *p* < 0.05.

## 3. Results

### 3.1. Pet Ownership during COVID-19

**Figure 1:**
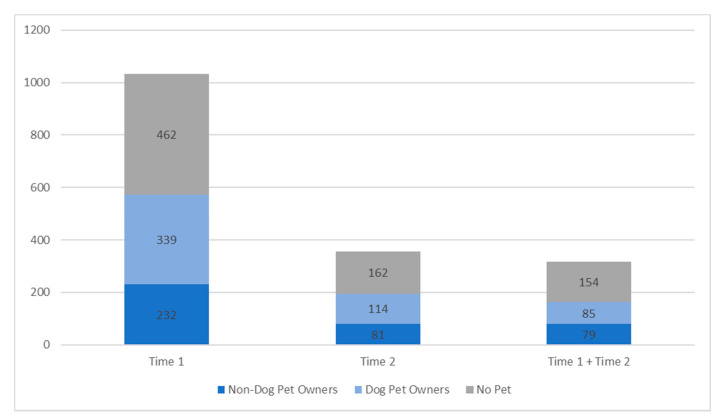
Proportion of pet owners for the cross-sectional samples (Time 1 only and Time 2 only) and for the longitudinal sample (Time 1 + 2).

At Time 1, 55.3% (*n* = 571) participants had a pet, and of those, 59.4% (*n* = 339) had a dog. At Time 2, 54.2% (*n* = 195) participants had a pet, and of those, 58.5% (*n* = 114) had a dog. Of the 318 participants who had data for both time points, 164 (51.6%) had a pet at both time points, 121 (38.1%) did not have a pet at either time point. Some of these participants lost (3.5%) or acquired (3.8%) a pet between Time 1 and Time 2; 10 participants had missing data for pet ownership. Of the 164 participants with a pet at both time points, 85 of them (55.9% of pet owners, 26.7% of overall sample) had a dog at both time points. See Figure 1 for pet ownership proportions.

Participants reported having an average of 1.72 pets (*SD* = 1.00, range 1 to 4 or more). At Time 2, the majority of youth reported that their family had obtained their pet over a year ago (*n* = 117, 66.5%), with 21% (*n* = 37) acquiring the pet within the last year, and 13% (*n* = 22) within the last three months (during the COVID-19 pandemic). Pet owning adolescents reported spending significantly more time with their pets during the pandemic (Time 2 *M* = 3.76, *SD* = 1.74) as compared to pre-COVID-19 (Time 1 *M* = 3.48, *SD* = 1.70), *t*(160) = −2.00, *p* = 0.047.

### 3.2. Pets and Loneliness

Cross-sectional analyses at Time 1 demonstrated no statistically significant association between pet ownership and loneliness, controlling for gender, age, and free/reduced price lunch status (see Table 1 and Table 2). There was a small effect of pet ownership on loneliness in Time 2, with pet owners reporting higher levels of loneliness than non-pet owners. Among pet owners, dog owners reported significantly lower levels of loneliness compared to non-dog pet owners at Time 1 (*p* = 0.025), but not during COVID-19 at Time 2 (*p* = 0.59). Although gender was a significant predictor of loneliness at both time points, there were no significant interactions between pet ownership and gender (Time 1: β = 0.05, *p* = 0.40, 95% CI [−0.08, 0.19]; Time 2: β = 0.05, *p* = 0.66, 95% CI [−0.21, 0.33]) or dog ownership and gender (Time 1: β = −0.01, *p* = 0.94, 95% CI [−0.19, 0.17]; Time 2: β = 0.21, *p* = 0.22, 95% CI [−0.15, 0.66]).

For youth who participated at both time points, pet owners reported higher increases in loneliness from Time 1 to Time 2 (pre-COVID-19 to during COVID-19) compared to non-pet owners (*p* = 0.008). Within pet owners, there was no significant difference between dog owners and non-dog pet owners (*p* = 0.27; see Table 3 for full regression results) on change in loneliness. Although gender was a significant covariate, there were no significant interactions between gender and pet ownership (β = 0.10, *p* = 0.39, 95% CI [−0.14, 0.35]) or dog ownership (β = 0.14, *p* = 0.46, 95% CI [−0.23, 0.51]) in predicting change in loneliness.

### 3.3. Pet Attachment

Pet attachment differed significantly by species, with dog owners reporting higher levels of attachment compared to non-dog pet owners (see Table 4 for full regression results). Gender was a significant predictor at Time 1, but there was no significant interaction between gender and dog ownership in predicting attachment at Time 1 (β = −0.11, *p* = 0.17, 95% CI [−0.56, 0.10]).

Time spent with a pet was also correlated with overall pet attachment at both Time 1 (*r* = 0.39, *p* < 0.001) and Time 2 (*r* = 0.37, *p* < 0.001). Among pet owners in the longitudinal sample, there was stability in pet attachment from Time 1 (*M* = 3.21, *SD* = 0.99) to Time 2 (*M* = 3.33, *SD* = 0.95), *t*(159) = −1.57, *p* = 0.12. Similarly, there was no change in attachment from Time 1 to Time 2 among dog owners (Time 1 *M* = 3.53, *SD* = 0.75, Time 2 *M* = 3.54, *SD* = 0.75, *t*(84) = −0.04, *p* = 0.97). Pet attachment was also not predictive of change in loneliness from Time 1 to Time 2 (see Table 5).

### 3.4. Pets and Coping with Stress

As expected, pet owners reported spending time with their pets as a stress-coping strategy more often than non-pet owners at both time points (See Table 6). For pet owners, spending time with pets was one of the highest rated stress coping strategies reported by participants. Other than spending time with a pet, pet owners and non-pet owners did not differ on any other stress-coping strategies at Time 1. However, at Time 2, pet owners were less likely to spend time with family or exercise/participate in sports as stress-coping strategies and more likely to spend time alone as a stress coping-strategy, as compared to non-pet owners. However, within pet owners in the longitudinal subsample, there was no significant change in spending time with a pet as a stress-coping strategy between Time 1 (*M* = 3.21, *SD* = 0.82) and Time 2 (*M* = 3.28, *SD* = 0.93), *t*(134) = −0.81, *p* = 0.42.

Spending time with a pet to cope with stress was significantly correlated with loneliness at Time 1 (*r* = 0.13, *p* = 0.005), suggesting that pet owners who were more lonely were also more likely to report turning to their pet when stressed. However, this correlation was small in magnitude and did not exist at Time 2 (*r* = 0.04, *p* = 0.571). Within pet owners, there were no differences between dog owners and non-dog pet owners in spending time with a pet as a stress-coping strategy at either Time 1 or Time 2 (Table 7).

## 4. Discussion

The results from this study did not support the presence of a buffering effect of human-animal interaction on loneliness for adolescents during the COVID-19 pandemic. However, the findings do indicate complexity in the relationships between pet ownership, attachment, loneliness, and coping with stress, and suggest that there may be species differences between dog owners and non-dog pet owners in these relationships. These results suggest a need for additional research exploring these complexities in more detail, further assessing how features of the relationship such as species and relationship quality might contribute to adolescent loneliness and stress coping strategies.

The results related to loneliness do not suggest a clear and consistent pattern of relationships between pet ownership, species of pet, and loneliness. Pet ownership was not a significant predictor of loneliness before COVID-19, but predicted higher levels of loneliness during the pandemic (although this effect was small in magnitude). However, dog ownership was predictive of lower levels of loneliness prior to COVID-19, but not during COVID-19. Interestingly, pet ownership more generally predicted higher increases in loneliness from before to during COVID-19, but there were no differences between dog owners and non-dog pet owners in these increases. These findings contrast with other recent research with adults in the UK that found smaller increases in loneliness during the pandemic for pet owners [17]. Pet relationships affect individuals in different ways; some individuals may benefit from pet ownership during the pandemic whereas others may find it more stressful to be a pet owner during this time. For example, there may be differences in pre-existing social networks between dog owners and non-dog owners that could have been disrupted during the pandemic. Continued research on pets and social support during the pandemic should explore the full breadth of individual and familial social networks to more accurately assess the specific impact of pet relationships within these networks.

While there is no theoretical reason to believe that pets would cause higher levels of loneliness for youth, these results point to the importance of looking at selection effects regarding who chooses to own pets. A fundamental challenge to human-animal interaction (HAI) research is the difficulty in parsing the effects of pet ownership from the factors contributing to an individual’s likelihood to own a pet. For example, it is still a largely unanswered question whether pet ownership can reduce loneliness, or whether individuals who are more or less lonely may be likely to acquire a pet (or a combination of both), and prior research has suggested that confounding demographic and contextual variables significantly complicate the relationship between pet ownership and health outcomes [27]. Furthermore, this issue becomes more complicated when exploring adolescent-pet relationships, as the degree to which an adolescent is involved in the decision to acquire a pet likely varies significantly across families. Nevertheless, the findings from this study are somewhat in line with some prior research demonstrating that pet owners often demonstrate poorer mental health compared to non-pet owners [28,29], which may be related to choosing to get a pet as a strategy for bolstering social support. As previously noted, much of the existing research on pets during the pandemic (as well as pets and loneliness) focuses on adult populations, and adolescents may be facing unique social effects associated with the pandemic that complicate these relationships and warrant further study.

As expected based on past research [22,30], dog owners did report being more highly attached to their pets than non-dog pet owners at both time points, and these differences in attachment were stable over time. These findings may help explain the differences in pre-COVID-19 loneliness for dog owners. If youth relationships with dogs involve higher levels of attachment and therefore a more socially supportive relationship, this relationship may contribute to lower levels of loneliness. However, similar to other recent research on pet interactions [16], in our study pet attachment more generally did not significantly predict changes in loneliness from before to during COVID-19. It is very likely that the effects of the pandemic on loneliness were significant enough to outweigh any potential benefits of dog ownership.

As demonstrated by the results regarding stress coping strategies, pet owning adolescents report spending time with a pet as a highly rated stress-coping strategy. Pet owners were also more likely to report spending time alone and less likely to use other proactive strategies such as spending time with family and exercising during COVID-19. These differences did not exist prior to COVID-19, perhaps suggesting a shift in stress-coping strategies during the pandemic. Prior to COVID-19 (but not during the pandemic), loneliness was positively correlated with spending time with a pet as a stress coping strategy, which suggests that youth who were lonely were more likely to turn to their pet when stressed and provides further support for a change in stress coping strategies from before to during the pandemic. Although the frequency of relying on a pet for social support did not differ from Time 1 to Time 2, this may have been due in part to a ceiling effect with pet contact being highly rated at both time points, especially given that there was a reported increase in time spent with the pet during the pandemic. Within the suite of strategies that youth may use for stress-coping, it appears that pets play a significant role that should be explored in more detail.

Another potential explanation for the mixed findings regarding any buffering effect of pet ownership is the potential for increased stressors such as financial strain related to pet ownership during the pandemic. Several studies of HAI during COVID-19 have suggested that pets may contribute to challenges during the pandemic, including being a potential barrier to accessing healthcare due to challenges with pet care during treatment [31] and demonstrating increased behavioral problems potentially due to increased stress and changes in owners’ routines during the pandemic [1]. However, the degree to which financial pressure is relevant to adolescents may vary by family context and age of the adolescent.

### Limitations and Future Directions

This study focused on attachment and stress-coping as processes that might contribute to the relationship between pet ownership and loneliness and did not address other possible mechanisms such as the provision of physical touch and reciprocity that could be particularly important in addressing the in-person social isolation associated with the COVID-19 pandemic [32]. It may be that some of the unique effects of dog relationships could be due to activities that are specific to dog ownership [33]. For example, Oliva and Johnston [16] found that dog owners reported that their dogs provided a reason to leave the house and socialize with other people during the pandemic. The measure of pet attachment and relationship quality we used in this study did not focus on specific attachment styles per se, and exploring different profiles of attachment styles and how they relate to outcomes such as loneliness may provide further insight into the nature of these relationships. Furthermore, the dichotomous predictors of pet ownership and dog ownership reduce our ability to detect nuance in these relationships. Future work should explore the diverse ways that adolescents interact with their pets and if certain types of interactions are related to social support, social isolation, and loneliness.

The proposed study leverages a diverse existing longitudinal dataset that allows for comparing pre- and during-COVID-19 outcomes. However, there are several limitations to the data, such as the significant attrition in the longitudinal sample from Time 1 to Time 2. It is possible that participants who were not able to be re-contacted at Time 2 had different challenges and limitations resulting from the pandemic compared to those who did participate. Within Time 1, participants with missing data differed systematically on several demographic variables, indicating that some missing data may not have been missing at random, limiting interpretability. Findings may also be limited by regression to the mean effects. In addition, Time 2 data were collected early in the pandemic. For some individuals this was the height of fear and uncertainty about the changes it would bring. Planned longitudinal follow-up with this sample will provide useful information about how adolescents may have adjusted to the long-term social impacts of the pandemic, and how pets fit into changing chronic stressors over time.

In addition, one of the primary outcomes of this study is loneliness, and recent research related to human-animal interaction and loneliness has suggested that typical indices of loneliness are not necessarily applicable to the specific ways in which pet relationships may buffer loneliness [34]. Future research should therefore use more nuanced assessments of loneliness to understand the potential benefits of pet ownership for individuals. Participants were also asked to report on their relationships with their favorite pet which limits our understanding of the ecosystem of pet ownership in the family if there are multiple pets. This limitation could potentially impact differences between species on the measured outcomes and does not allow us to assess if there are any cumulative effects of having multiple pets.

Overall, HAI research is challenged by the significant complexities in the diverse relationships that individuals have with their pets, and the various methodological and measurement approaches used to assess these relationships [35]. For future research measuring the interaction between pet relationships and loneliness, there appears to be a need for more nuanced methodology and measurement approaches that account for individual and family-level differences in how pet ownership contributes to resilience during times of stress. Our results also point to the need for assessing species-level differences, selection effects with regard to pet ownership, and how quality of relationship contributes to socially-oriented outcomes.

## 5. Conclusions

This study assessed the relationship between pet ownership, pet attachment, loneliness, and coping with stress before and during the COVID-19 pandemic. Results indicated that there was not a buffering effect of pet ownership on loneliness, and that pet ownership was in fact predictive of increases in loneliness from pre-pandemic to during the pandemic. Dog owners showed lower levels of loneliness prior to the pandemic as well as higher levels of attachment, suggesting possible species-level differences in these relationships. Pet owners also reported spending time with their pet as a highly used strategy for coping with stress, suggesting that future research should explore the role of pets in coping with stress and social isolation during the pandemic. Considering these findings, it is important to recognize that the impact of the pandemic on youth may be more powerful than any single coping strategy. Therefore, pet relationships should be explored within the context of other social and relational sources of support and not treated as a stand-alone solution for ameliorating the negative social impacts of the pandemic.

## Figures and Tables

**Table 1:** Pet ownership and dog ownership predicting loneliness at Time 1 (pre-COVID) and Time 2 (during COVID), controlling for gender, age, and free/reduced price lunch status.

	Loneliness Time 1 (*n* = 910)					Loneliness Time 2 (*n* = 310)				
	B (SE)	β	*p*	*sr*	95% CI	B (SE)	β	*p*	*sr*	95% CI
Pet Ownership	−0.05 (0.03)	−0.05	0.14	−0.05	−0.12–0.02	0.13 (0.06)	0.12	0.039	0.12	0.01–0.26
Female	0.20 (0.03)	0.19	<0.001	0.19	0.13–0.26	0.16 (0.07)	0.14	0.017	0.14	0.03–0.30
Age	0.03 (0.01)	0.06	0.08	0.06	−0.00–0.05	0.01 (0.03)	0.02	0.684	0.02	−0.05–0.07
Free/Reduced-price lunch	0.08 (0.04)	0.07	0.05	0.07	0.00–0.17	0.17 (0.08)	0.12	0.032	0.12	0.01–0.33
	**Loneliness Time 1 (*n* = 514)**					**Loneliness Time 2 (*n* = 173)**				
	**B (SE)**	**β**	***p***	***sr***	**95% CI**	**B (SE)**	**β**	***p***	***sr***	**95% CI**
Dog Ownership	−0.10 (0.05)	−0.10	0.025	−0.10	−0.19–0.01	−0.05 (0.10)	−0.04	0.594	−0.05	−0.24–0.14
Female	0.22 (0.04)	0.21	<0.001	0.21	0.13–0.31	0.19 (0.10)	0.15	0.055	0.15	−0.01–0.39
Age	0.02 (0.02)	0.05	0.27	0.05	−0.02–0.06	<0.01 (0.04)	0.01	0.877	0.01	−0.08–0.09
Free/Reduced-price lunch	0.07 (0.06)	0.05	0.25	0.05	−0.05–0.18	0.19 (0.12)	0.11	0.138	0.11	−0.06–0.43

Note: sr = semi-partial correlation; B = unstandardized beta.

**Table 2:** Loneliness at Time 1 (pre-COVID) and Time 2 (during COVID), by pet ownership status.

	Loneliness Time 1		Loneliness Time 2	
	*M*	*SD*	*M*	*SD*
Pet Owners	1.43	0.52	1.62	0.60
Non-Pet Owners	1.47	0.53	1.49	0.52
Dog Owners	1.38	0.47	1.60	0.57
Non-Dog Pet Owners	1.50	0.59	1.65	0.64

**Table 3:** Pet ownership and dog ownership predicting Time 2 loneliness, controlling for Time 1 loneliness, gender, age, and free/reduced price lunch eligibility.

	**Pet Owners vs. Non Pet Owners (*n* = 252)**				
	**B (SE)**	**β**	***p***	***sr***	**95% CI**
Pet Ownership	0.16 (0.06)	0.14	0.008	0.14	0.04–0.27
Time 1 Loneliness	0.53 (0.05)	0.53	<0.001	0.53	0.43–0.63
Female	0.14 (0.06)	0.12	0.02	0.12	0.02–0.26
Age	0.02 (0.03)	0.04	0.43	0.04	−0.03–0.08
Free/Reduced-price lunch	0.11 (0.08)	0.08	0.13	0.08	−0.03–0.26
	**Dog Owners vs. Non-Dog Pet Owners (*n* = 134)**				
	**B (SE)**	**β**	***p***	***sr***	**95% CI**
Dog Ownership	0.10 (0.09)	0.08	0.28	0.08	−0.08–0.28
Time 1 Loneliness	0.60 (0.08)	0.57	<0.001	0.54	0.45–0.76
Female	0.21 (0.09)	0.16	0.03	0.16	0.02–0.39
Age	0.03 (0.04)	0.06	0.44	0.06	−0.05–0.11
Free/Reduced-price lunch	0.09 (0.12)	0.06	0.44	0.06	−0.14–0.32

Note: sr = semi-partial correlation; B = unstandardized beta.

**Table 4:** Dog ownership (vs. non-dog pet owners) predicting pet attachment at Time 1 (pre-COVID) and Time 2 (during COVID).

	Attachment Time 1 (*n* = 545)					Attachment Time 2 (*n* = 190)				
	B (SE)	β	*p*	*sr*	95% CI	B (SE)	β	*p*	*sr*	95% CI
Dog Ownership	0.35 (0.08)	0.18	<0.001	0.18	0.18–0.51	0.49 (0.14)	0.25	<0.001	0.25	0.22–0.76
Female	0.19 (0.08)	0.10	0.03	0.10	0.03–0.35	0.23 (0.15)	0.11	0.13	0.11	−0.07–0.52
Age	−0.04 (0.03)	−0.05	0.26	−0.05	−0.10–0.03	−0.06 (0.07)	−0.07	0.33	−0.07	−0.19–0.07
Free/Reduced-price lunch	0.17 (0.11)	0.07	0.10	0.07	−0.03–0.38	0.11 (0.18)	0.04	0.55	0.04	−0.25–0.47

Note: sr = semi-partial correlation; B = unstandardized beta.

**Table 5:** Pet attachment predicting Time 2 loneliness, controlling for Time 1 loneliness.

	B (SE)	β	*p*	*sr*	95% CI
Pet Attachment	−0.01 (0.05)	−0.02	0.84	−0.01	−0.10–0.08
Time 1 Loneliness	0.59 (0.08)	0.55	<0.001	0.52	0.44–0.75
Female	0.15 (0.09)	0.11	0.12	0.11	−0.04–0.33
Age	0.04 (0.04)	0.06	0.36	0.06	−0.04–0.12
Free/Reduced-price lunch	0.01 (0.12)	0.01	0.93	0.01	−0.22–0.24

Note: sr = semi-partial correlation; B = unstandardized beta.

**Table 6:** Average frequency of use of stress coping strategies at Time 1 (pre-COVID) and Time 2 (during COVID), stratified by pet ownership status.

	Coping Strategies Time 1						Coping Strategies Time 2					
	Pet Owners		Non-Pet				Pet Owners		Non-Pet			
	*M*	*n*	*M*	*n*	*t*	*p*	*M*	*n*	*M*	*n*	*t*	*p*
Spending time with pet(s)	3.07	495	2.29	191	−8.64	<0.001	3.29	171	1.82	57	−8.97	<0.001
Being alone	2.46	490	2.56	377	1.42	0.156	3.02	171	2.74	136	−2.28	0.023
Spending time with family	2.86	501	2.94	377	1.28	0.203	2.63	170	2.87	135	2.04	0.042
Spending time with a close friend	3.17	497	3.22	376	0.80	0.422	3.33	170	3.40	131	0.68	0.498
Posting about it on social media	1.56	472	1.56	331	0.02	0.987	1.32	161	1.26	110	−0.75	0.455
Watching my favorite movies or shows	2.98	496	3.08	371	1.66	0.097	3.18	168	3.17	125	−0.15	0.879
Exercising or sports	2.70	493	2.77	356	1.03	0.302	2.69	169	2.95	125	2.10	0.037
Playing video or online games	2.52	495	2.61	355	1.26	0.210	2.59	166	2.71	121	0.86	0.393
Spending time outdoors or in nature	2.73	495	2.83	366	1.50	0.135	2.99	168	3.12	130	1.16	0.246
Creating video content for social media (e.g., Tik Tok)	Time 2 Only		Time 2 Only		-	-	1.98	163	1.78	110	−1.53	0.126
Video hangouts (e.g., Zoom, Google, Skype etc.)	Time 2 Only		Time 2 Only		-	-	2.03	172	2.11	128	0.62	0.534

**Table 7:** Dog ownership (vs. non-dog pet ownership) predicting spending time with a pet as a stress-coping strategy, controlling for gender, age, and free/reduced-price lunch status.

	Spending Time with Pet Time 1 (*n* = 150)					Spending Time with Pet Time 2 (*n* = 144)				
	B (SE)	β	*p*	*sr*	95% CI	B (SE)	β	*p*	*sr*	95% CI
Dog Ownership	0.15 (0.14)	0.09	0.295	0.09	−0.13–0.43	0.13 (0.16)	0.07	0.417	0.07	−0.19–0.46
Female	−0.27 (0.15)	−0.15	0.070	−0.15	−0.57–0.02	<0.01 (0.17)	0.00	0.999	0.00	−0.34–0.34
Age	0.05 (0.06)	0.07	0.428	0.07	−0.08–0.18	0.04 (0.08)	0.04	0.643	0.04	−0.11–0.18
Free/Reduced-price lunch	0.02 (0.20)	0.01	0.915	0.01	−0.37–0.41	−0.18 (0.21)	−0.07	0.405	−0.07	−0.60–0.24

Note: sr = semi-partial correlation; B = unstandardized beta.

## Data Availability

The data presented in this study are available on request from the corresponding author. The data are not currently publicly available due to ongoing data collection for the full longitudinal study.

## Supplementary Materials

The following are available online at https://www.mdpi.com/2076-2615/11/3/885/s1, Supplementary Table S1. Missingness by item, stratified by time point.
