# Supplementary material for: Companion Animal Relationships and Adolescent Loneliness during COVID-19
Source: Animals (Basel). 2021 Mar 19;11(3):885. doi: 10.3390/ani11030885 (PMC8003635; doi:10.3390/ani11030885)
Supplement: Supplementary file 1 [file animals-11-00885-s001.pdf]

Supplementary Material: Missing Data Analyses

Missing data was assessed by item, as indicated below in Supplementary Table 1, and ranged from 1% to 15%.

Scheme 1. Missingness by item, stratified by time point.

	Time 1 (*n* = 1033)				Time 2 (*n* = 360)			
	**Valid *n***	**Missing *n***	**% Missing**	**Total *n***	**Valid *n***	**Missing *n***	**% Missing**	**Total *n***
Gender	1018	15	1%	1033	353	7	2%	360
Age	1033	0	0%	1033	358	2	1%	360
Free/reduced price lunch	1013	20	2%	1033	349	11	3%	360
Pet ownership	1021	12	1%	1033	346	14	4%	360
Dog ownership	571	0	0%	571	195	0	0%	195
Pet Attachment scale	555	16	3%	571	192	3	2%	195
Loneliness scale	930	103	10%	1033	314	46	13%	360
**Coping with Stress**								
Being alone	900	133	13%	1033	309	51	14%	360
Spending time with family	910	123	12%	1033	308	52	14%	360
Spending time with a close friend	908	125	12%	1033	310	50	14%	360
Spending time with pet(s)	905	128	12%	1033	309	51	14%	360
Posting about it on social media	910	123	12%	1033	310	50	14%	360
Watching my favorite movies or shows	908	125	12%	1033	310	50	14%	360
Exercising or sports	907	126	12%	1033	310	50	14%	360
Playing video or online games	907	126	12%	1033	307	53	15%	360
Spending time outdoors or in nature	908	125	12%	1033	309	51	14%	360
Creating video content for social media	n/a				309	51	14%	360
Video hangouts	n/a				310	50	14%	360

*Note Coping with Stress valid responses include "Does not apply to me"

In addition, participants who did not complete the outcomes ("non-completers") were compared to those who completed one or more outcomes ("completers") on demographic variables and pet ownership using chi-square analyses. At Time 1, there was a higher percentage of non-completers who received free/reduced price lunch compared to completers ($p = 0.001$). There were also differences between grade levels ($p = 0.005$), with a higher percentage of 6th grade non-completers and a lower percentage of 9th grade non-completers as compared to completers. Pet ownership ($p = 0.11$) and gender ($p = 0.07$) were not predictive of missigness at Time 1. These results indcate that data may not have been missing completely at random at Time 1. At Time 2, there were no sigificant differences between completers and non-completers on pet ownership, gender, free/reduced price lunch status or grade level (all $p > 0.05$). These results suggest that data were missing at random at Time 2.
